# Acute Acalculous Cholecystitis Induced by Acute Hepatitis B Virus Infection

**DOI:** 10.1155/2012/132345

**Published:** 2012-12-12

**Authors:** Riyadh Ali Mohammed, Wisam Ghadban, Osama Mohammed

**Affiliations:** ^1^Department of Medicine, Hamad Medical Corporation, Doha, Qatar; ^2^Department of Accident & Emergency, Hamad Medical Corporation, Doha, Qatar

## Abstract

During the course of acute viral hepatitis, some functional and anatomical changes to the gallbladder can occur. Acute acalculous cholecystitis (ACC) is a rare complication of acute hepatitis B virus infection; only few cases are reported as ACC associated with acute hepatitis B virus infection. ACC cases are self-limiting, while other limited cases can progress to a gangrenous state, perforation, and even death. We present a 27-year-old female case diagnosed to have acute acalculous cholecystitis and associated with acute hepatitis B virus infection, and she recovered within one week of her presentation without complication or surgical intervention.

## 1. Background

During the course of acute viral hepatitis, some functional and anatomical changes to the gallbladder can occur. Acute acalculous cholecystitis (ACC) is a rare complication of acute viral hepatitis. Some of these cases are self-limiting, while other limited cases can progress to a gangrenous state, perforation, and even death.

The definition of ACC is the inflammation of gallbladder wall in the absence of calculous or sludge; the incidence of ACC is about 5–15% of all cases of cholecystitis, of which 47% occur after surgical procedure, and the other percentage due to prolonged immobilization, starvation for a long time, and sepsis [1–3]. The pathogenesis of acalculous cholecystitis occurred because of the detergent effect of bile on the epithelium, ischemic injury of the gallbladder epithelium; immune complex deposits in the vessel wall of gallbladder may cause necrotizing vasculitis as an extrahepatic complication of chronic hepatitis B virus (HBV) infection [4]. Some cases of HAV reported that the pathogenesis of ACC is due to direct invasion of the virus to gallbladder wall [5, 6]. 

During the course of acute hepatitis, gallbladder wall oedema and slowing of bile clearance which may lead to the formation of bile sludge and thickening of the gallbladder wall [7–9]. Limited number of cases of ACC were reported to be associated with acute viral hepatitis A infection [5, 10–13] and only one case reported with hepatitis B infection [14].

ACC associated with acute viral hepatitis can present in different conditions; most cases are self-limiting, while limited number of cases progress to a gangrenous state, gallbladder perforation, and sometimes death [5, 12, 13]. We would like to present here a second reported case of ACC associated with an acute hepatitis B virus infection [14].

## 2. Case Report

This is a 27-year-old female patient, who came to A&E with 9-day history of epigastric pain, nausea, anorexia, and generalized fatigue. The pain increased over the last 5 days with the radiation to right hypochondriac area and to the upper back and increased after meal with yellowish discoloration of sclera and dark color urine; history of occasional attacks of vomiting, no history of fever, and no past medical illnesses were reported, her husband has chronic HBV infection recently diagnosed but not on treatment (planned to start treatment within next few weeks). Upon physical examination her sclera is icteric; abdominal examination revealed epigastric and right hypochondriac area tenderness, liver palpable two fingers below costal margin. Blood investigations showed normal complete blood count, normal electrolytes, normal urea, and creatinine.

Alanine aminotransferase (ALT) 2488 u/L (norml 0–31) and aspartate aminotransferase (AST) 1590 u/L (normal 0–30), total bilirubin 63 umol/L and direct bilirubin 52 umol/L, alkaline phosphatase (ALP) 152 u/L, and amylase and lipase are normal; total protein 70 g/L and albumin is 41 gm/L, her prothrombin time (PT) is 18.8 second, INR is 1.7, partial thromboplastin time (PTT) is 25.6 second, WBC is 6300, Hb is 12.9 gm/dL, platelets are 172,000, ESR is 42, CRP is 65, hepatitis B surface Ag is positive, hepatitis B core antibody IgM is positive, hepatitis B e antigen and antibody are positive, and hepatitis A IgM and hepatitis C virus antibody are negative. 

**Figure 1:**
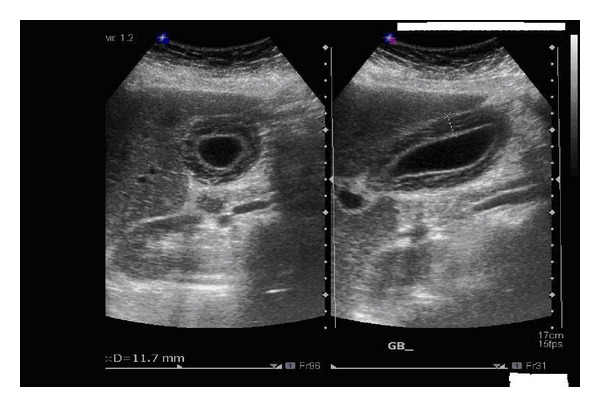
U/S of abdomen showing the thickened gallbladder wall.

Abdominal ultrasound (Figure 1) on admission revealed mild hepatosplenomegaly, and gall bladder showed diffuse thickening and stratification of wall (11.7 mm), no calculi/pericholecystic fluid, normal CBD (4.8 mm), and free fluid along subhepatic region as well as right iliac region.

The patient was treated with supportive therapy of intravenous (I.V.) fluid, I.V. metoclopramide, and I.V. ranitidin with low fat and high carbohydrate diet.

During the followup, her symptoms of abdominal pain and nausea improved gradually, while vomiting and jaundice have settled completely by day 5 and her investigations showed the following: ALT 595 u/L (normal 0–31), AST 77 u/L (normal 0–30), ALP 118 u/L, total bilirubin 22 umol/L, PT 13.4 second, INR 1.1, and PTT 28 second; HBV DNA viral load was done later with lower limit of detection. 

**Figure 2:**
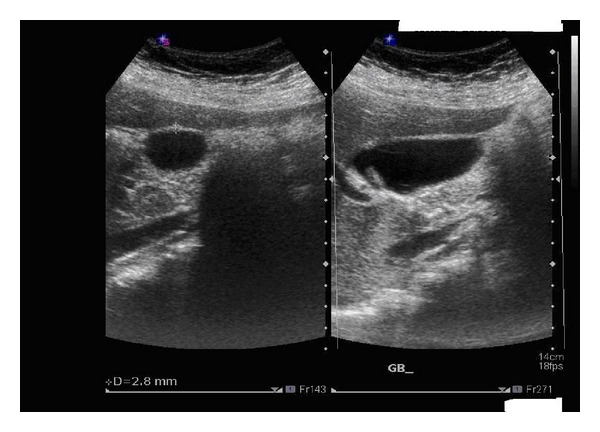
Abdominal U/S showing normal thickness of gallbladder wall.

Abdominal U/S was repeated on day 5 of admission (Figure 2) and revealed normal size gallbladder with no calculi and wall thickness about 2.8–3 mm, bulky fatty liver.

The patient was discharged with good general condition and a medical clinic appointment for followup was given.

## 3. Discussion

Acute acalculous cholecystitis is the inflammation of the gallbladder wall in the absence of stone or sludge [15], so the diagnostic criteria for ACC are well documented in this case as the patient has right hypochondriac pain and tenderness, pain radiated to the back and increased after food, abdominal U/S imaging of thickened gallbladder wall (more than 4 mm), and the absence of ascites or hypoalbuminemia and there is no calculi or sludge.

The pathogenesis of ACC is correlated with the ischemic injury of GB epithelium. A review of 15 ACC cases proposes that the overactivation of factor XII-dependent coagulation pathway triggers the injury of vessels that are located in the muscular and serosal layers of GB wall [1], and if it continues, it will lead to ischemia and finally necrosis. The GB wall epithelium has high energy requirements in order to maintain its high metabolic activity. Immobilization, starvation for a long time, and sepsis lead to splenic vasoconstriction and cause GB epithelium to be more vulnerable to ischemic injury [16]. Hakala et al. reported that when they compared the surgical specimens of patients suffering from ACC and calculous cholecystitis, they noticed a marked decrease in vascular filling of the GB in the ACC patients [17].

Another pathogenesis for ACC is the detergent effect of bile on the GB epithelium. GB stores and concentrates the bile and in response to cholecystokinin (CCK), which is secreted after the meal, contracts and releases the concentrated bile into the duodenum. These physiological steps prevent the detrimental effect of bile on GB epithelium. In the situation of long-lasting starvation, because of the lack of CCK secretion, GB fails to release the concentrated bile into the duodenum and this gives rise to epithelial injury [16, 18]. 

The 3rd possible pathogenesis is related to the possibility of the direct viral invasion to the biliary tract and the gallbladder wall as reported in some cases with acute hepatitis A virus infection [5, 6]. 

Necrotizing vasculitis plays a role in the possible pathogenesis of ACC in association with hepatitis B virus infection as diagnosed by histopathology for a case with chronic hepatitis B virus infection [4]. Approximately 20% of acute hepatitis B patients develop extrahepatic complications [19]. The most common extrahepatic complication is polyarthritis, which precedes the onset of jaundice. The inhibition of viral replication causes both biochemical and clinical resolution in acute viral hepatitis [19]. This fact drives us to consider a close relationship between the pathogenesis of extrahepatic complications and the immune complexes. Necrotizing vasculitis is usually related to polyarteritis nodosa (PAN), which affects small and medium diameter arteries in HBV-positive patients.

In conclusion functional and structural changes to GB can occur during the course of acute viral hepatitis; however ACC is an extremely rare complication of acute viral hepatitis, and the mortality from ACC with viral hepatitis is extremely low in comparison to ACC of other origin that needs urgent surgical intervention, hence clinical followup and observation may be adequate in selected patients, so we can avoid unnecessarily invasive procedure.
